# An Unusual Cause of Left-sided Severe Chest Pain

**DOI:** 10.4103/1319-3767.74445

**Published:** 2011

**Authors:** Vipul D. Yagnik, Bhargav D. Yagnik

**Affiliations:** Department of Surgery, Ronak Endo-Laparoscopy and General Surgical Hospital, Patan, India; 1 General Practitioner, Ahmedabad, Gujarat, India

A 50-year-old lady presented to the emergency room with severe left-sided chest pain following an episode of forceful vomiting. A history of giddiness was present. Her past medical and surgical history were insignificant. On examination, she was having a respiratory rate of 24/min and her blood pressure was 100/60 mmHg. She was diaphoretic and surgical emphysema was present. On plain radiography, hydropneumothorax was present. Gastrograffin study of the patient was performed Figure 1.

**Figure 1:**
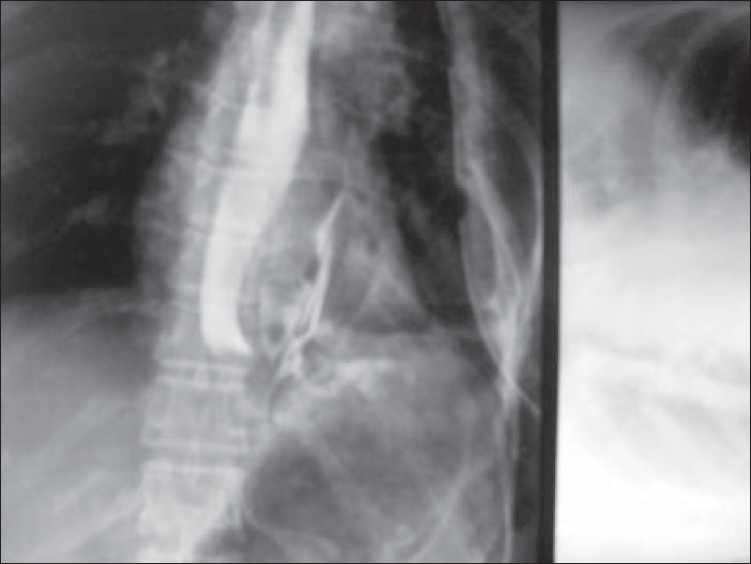
Gastrograffin study

## QUESTIONS

Q1. What is the diagnosis?
Q2. What is the classical presentation of this condition?
Q3. What are the characteristics of pleural fluid in this condition?
Q4. Which are the factors that decide the line of management in this condition?
Q5. What are the indications for conservative line of management in this condition?

## ANSWERS

A1. Gastrograffin study revealed leak of the contrast into the left posterolateral aspect of pleural cavity, which indicates perforation in the esophagus. This condition is known as Boerhaave syndrome.
A2. A classical presentation of this condition is Mackler triad: vomiting, subcutaneous emphysema, and lower thoracic pain.
A3. The characteristics of pleural fluid include a pH of <6, undigested food particles, and a high amylase content.[1]
A4. Location of perforation, etiology, and time interval between injury and diagnosis are the factors that decide the line of management.[2]
A5. Indications for conservative management include the following:[3] absence of clinical signs of infection, perforation that drains back into the esophagus, and contained perforation within the thoracic cavity.
